# Traffic Speed Data Imputation Method Based on Tensor Completion

**DOI:** 10.1155/2015/364089

**Published:** 2015-03-03

**Authors:** Bin Ran, Huachun Tan, Jianshuai Feng, Ying Liu, Wuhong Wang

**Affiliations:** ^1^Department of Civil and Environmental Engineering, University of Wisconsin-Madison, Madison, WI 53706, USA; ^2^Department of Transportation Engineering, Beijing Institute of Technology, Beijing 100081, China; ^3^College of Computer and Information Engineering, Beijing Technology and Business University, Beijng 100048, China

## Abstract

Traffic speed data plays a key role in Intelligent Transportation Systems (ITS); however, missing traffic data would affect the performance of ITS as well as Advanced Traveler Information Systems (ATIS). In this paper, we handle this issue by a novel tensor-based imputation approach. Specifically, tensor pattern is adopted for modeling traffic speed data and then High accurate Low Rank Tensor Completion (HaLRTC), an efficient tensor completion method, is employed to estimate the missing traffic speed data. This proposed method is able to recover missing entries from given entries, which may be noisy, considering severe fluctuation of traffic speed data compared with traffic volume. The proposed method is evaluated on Performance Measurement System (PeMS) database, and the experimental results show the superiority of the proposed approach over state-of-the-art baseline approaches.

## 1. Introduction

The large amounts of traffic data collected from the traffic sensors are extremely valuable for route guidance, planning, and management of Intelligent Transportation Systems (ITS) [1]. The data, which include traffic speed, volume, and occupancy, are collected via various traffic collecting devices and technologies. Traffic speed data is one of the most important information sources for ITS, Advanced Traveler Information Systems (ATIS), and Advanced Traffic Management Systems (ATMS) [1]. As one of important parameters of traffic data, traffic speed data play a prominent role in the traffic domain and convey more information on traffic state, such as traffic congestion, than traffic volume data. For example, traffic speed data are used for computing the traffic congestion index in Beijing. Moreover, the traffic speed data are used for the purpose of traffic guidance [2].

Despite the importance of traffic speed information, unfortunately, substantial missing data are usually induced due to various malfunctions in data collection and/or record systems, such as failed loop detectors, failed loop amplifiers, and failed signal communication and processing devices. However, most of traffic data analysis methods used in intelligent transportation systems require the completeness of data and thus missing traffic speed data will severely degrade the performance of ITS, such as the accuracy of travel time estimation. Besides improving the reliability of traffic data collection and record systems, the research on the problem of missing data in intelligent transportation systems has been aroused due to extensive concern by traffic engineers and researchers.

In the past decade, various imputation methods have been proposed for solving the missing traffic data problem. Among these methods, most of them focus on traffic volume data imputation, and only a few researches try to deal with missing traffic speed data estimation. Since traffic speed data have some similar characteristics with traffic volume data, such as temporal and spatial correlations, some imputing methods for missing traffic volume data can be applied directly to traffic speed data. However, compared with traffic volume data imputation, there are many special characteristics of speed data. One major observation for traffic speed data imputation is that traffic speed data fluctuate more severely compared with traffic volume data, which poses significant challenge to traditional methods.

In general, the frequently used methods include historical (neighboring) imputation methods [3] and spline (including linear)/regression imputation methods [4]. The historical imputation method fills a missing data point with a known data point collected on the same site at the same daily time but from a neighboring day [5]. A variation of this algorithm fills the missing data with the average values taken over the most recent days [6]. The spline/regression imputation method recovers the missing values by applying mathematical interpolation algorithms according to the surrounding known data points collected during the same day [7–9]. The imputing performance of above methods greatly depends on the surrounding data of missing points, and thus these methods fail to estimate the missing value at high missing ratio. Meanwhile, the above methods fail to capture the global information of data sets. As a result, this kind of methods will produce relative poor results for imputing missing speed data due to its high fluctuation characteristics.

Because of the above reason, researchers proposed Bayesian Principal Component Analysis (BPCA) algorithm [10] and Probabilistic Principal Component Analysis (PPCA) [7] for addressing missing data problem. BPCA is a modification of PPCA. Indeed, both PPCA and BPCA are based on EM imputation methods [11] and make use of the relationship between the observed data and the latent variables for imputing the missing data. Generally, the relationship between the observed data and the latent variables is described as probabilistic model. In order to obtain the maximum probability of above parameters, the Bayesian model is introduced to estimate the missing values with respect to the estimated posterior distribution. The missing values are gradually recovered, along with the building of the latent model. Intuitively, the two methods make a reasonable trade-off among the periodicity, local predictability, and other statistical property of the traffic flow. This ability helps them to outperform the traditional methods since they exploit more temporal correlations than traditional methods. Widhalm et al. [12] proposed a method based on Gaussian-mixture model to estimate road link speed from sparse or missing probe vehicle data. The traffic speed is estimated only from sparse (only a few available observations) historical data of all links in the road network. However, these methods may perform poorly when the missing ratio is high due to the intrinsic characteristic of EM method and the intrinsic characteristic of matrix model.

To improve the performance of traffic data imputation, tensor pattern is introduced to represent the traffic data while encoding multicorrelations in traffic data, and tensor-based imputation algorithms mine the multicorrelations of the constructed tensor while estimating the missing entries. Tan et al. [13] construct traffic volume data as a tensor model and propose an efficient algorithm based on tensor completion to impute missing traffic volume. It exploits global information of traffic volume data, specifically, tensor based model can exploit multiway global information simultaneously, such as temporal and spatial information. Though this method shows its superiority in traffic volume data imputation, the performances on estimating missing traffic speed data are not reported. Asif et al. [14] proposed a low-dimensional model to impute the missing speed data in road network. They model the traffic data as matrix and/or tensor and use Fixed Point Continuation with Approximate SVD (FPCA) [15] and Canonical Polyadic (CP) decomposition [16] to solve the problem of missing traffic speed data. The missing speed data can be imputed more accurately than tradition methods based on the multiway global information. However, most of them focused on traffic volume data imputation, and they did not discuss how to exploit the multiway global information of traffic speed data.

In this paper, we focus on the missing speed data imputation on freeway. Motivated by the work in [13], this paper adopts tensor pattern to model the traffic speed data, and then an efficient tensor completion method which can deal with noisy entries is used to estimate the missing traffic speed data due to the severe fluctuation of traffic speed data. The correlations of traffic speed data are analyzed firstly, and then tensor pattern is used for modeling traffic speed which could benefit for mining the underlying multimode correlations while keeping its natural structure. To estimate the missing entries in the traffic speed tensor, a high accuracy low rank tensor completion algorithm called HaLRTC [17] is adopted, which can deal with noisy observed entries. The proposed method is evaluated on the Performance Measurement System (PeMS) database (http://pems.dot.ca.gov/) and experimental results show the proposed method achieves higher recovery accuracy than the state-of-the-art missing traffic speed data imputation methods.

To give a detailed explanation, the rest of this paper is organized as follows.  Section 2 presents the notations used in this paper and introduces tensor completion algorithms for missing data estimation. The intrinsic correlations of traffic speed data, such as week-to-week relations, day-to-day relations, and hour-to-hour relations, are analyzed in Section 3. In Section 4, we propose a general model to describe traffic data tensor completion problem and give a high accuracy low rank tensor completion algorithm (HaLRTC) to solve it. Numerical experiments are reported in Section 5 followed by conclusion in Section 6.

## 2. Notations and Tensor Completion

In this section, the notations used in this paper are presented, and then the matrix and tensor completion algorithms for missing data estimation are introduced.

### 2.1. Notations

We denote the scalars in *ℛ* with lowercase letters (*a*, *b*,…) and the vectors with bold lowercase letters (**a**, **b**,…). The matrices are written as uppercase italic letters, for example, **X**, and the symbols for tensors are handwriting letters, for example, *𝒳*. The subscripts represent the following scalars: (*𝒳*)_*ijk*_ = *x*_*ijk*_, (**X**)_*ij*_ = *x*_*ij*_. The superscripts indicate the size of the matrices or tensors. For example, there is a set of traffic volume data which are recorded every 5 minutes for 16 days. Then, the data of one day preserves 288 data points (12 hours per day and 24 data points per hour). Therefore, the traffic data of 16 days can be constructed as a matrix model of size 16 × 288 or a tensor model of size 16 × 12 × 24. The Frobenius norm of matrix **X** is defined as ‖**X**‖_*F*_≔(∑_*i*,*j*_|*x*_*ij*_|^2^)^1/2^. Let Ω be an index set, then **X**_Ω_ denotes the vector consisting of elements in the set Ω only. Define ‖**X**‖_Ω_ = (∑_(*i*,*j*)∈Ω_ *x*_*ij*_^2^)^1/2^.

An *n*-way tensor can be rearranged as a matrix, this is called matricization, also known as unfolding or flattening a tensor. The “unfold” operation along the *n*th mode on a tensor *𝒳* of size *I*_1_ × *I*_2_ × ⋯×*I*_*N*_ is defined as unfold  (*𝒳*, *n*) = **X**_(*n*)_. The opposite operation “fold” is defined as fold  (**X**_(*n*)_) = *𝒳*. For example, the above tensor model *𝒳* for traffic volume data which of size 16 × 12 × 24 can be unfolded along the 1th mode, and get a matrix **X**_(1)_ of size 16 × 288. In addition, the mode-*n* rank of *𝒳* is denoted as rank_*n*_(*𝒳*), which is equal to the column rank of **X**_(*n*)_.

### 2.2. Tensor Completion Methods

The matrix and tensor completion methods were recently proposed for addressing the missing data problem. Those methods can perform well even when the missing ratio is very high.

During the past years, there were lots of works on matrix completions. Recently, most theoretical work focuses on proving bounds for the exact matrix completion problem, and a lot of work focuses on low rank or approximate low rank matrix completion problems. Candès and Recht [18] introduced a convex optimization to solve the matrix completion problem by modeling it as a Semi-Definite Programing (SDP). FPCA [15] and SVT [19] are the other two algorithms for solving the low-rank matrix completion problem. ADMiRA [20] is an iterative method for solving a least squares problem with the restriction of rank. OptSpace [21] is an efficient procedure to solve the exact and approximate matrix completion problems.

Tensor completion methods can be seen as a high-order extension of matrix completion methods. They can capture more global information than matrix completion methods due to the intrinsic multiway characteristics of tensor model. Liu et al. [17] first proposed a tensor completion method based on trace norm minimization and applied it on image completion. Also, a first-order method has been recently developed called CP-WOPT [16] base on CP decomposition of tensor model and applied on imputing missing network traffic data. Signoretto et al. [22] established a mathematical framework for learning with higher order tensors respect to missing data.

Traffic speed data have intrinsic multiway spatial-temporal correlations. For fully exploiting the spatial-temporal correlations and improving the performance of imputation methods, a multiway tensor model is utilized to construct the traffic speed data and a high accuracy low rank tensor completion algorithm is used to address the missing speed data in this paper.

## 3. Correlation Analysis of Traffic Speed Data

As mentioned above, the core idea of the approaches for addressing missing data problems was to make use of the established intrinsic relations among those data [23]. Exploiting the useful intrinsic information of traffic speed data attracts continuous interest due to its wide applications, especially in missing traffic speed data imputation.

**Figure 1:**
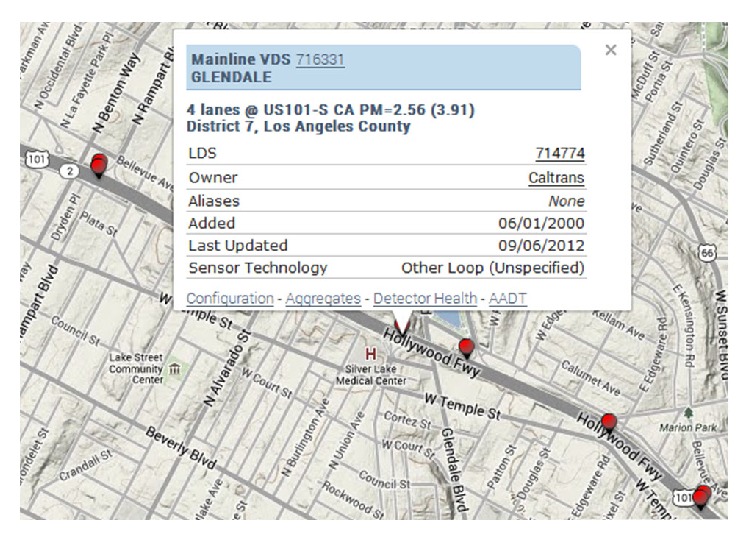
Loop Detectors in District 7, Los Angeles County.

Below we will illustrate the traffic speed data downloaded from PeMS (http://pems.dot.ca.gov/) and analyze the correlations between each mode of traffic speed data. We downloaded a set of traffic speed data in District 7, Los Angeles County (see Figure 1). The area covers 13 loop detectors for a direction. The traffic speed data are recorded by every 5 minutes. And the whole period of the data lasts for 28 days, that is, from February 4 to March 3, 2013.

**Figure 2:**
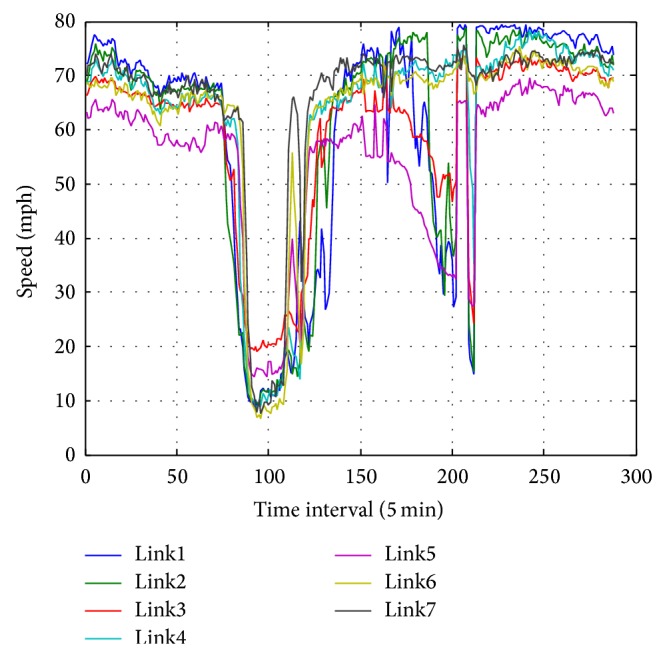
The daily profile of the traffic speed data on Monday for seven detectors.

For illustrating the spatial correlations of traffic speed data, seven adjacent detectors are chosen randomly from the above area for simplicity. And the data on Monday for each detector are plotted in Figure 2. The figure shows that the traffic speed data between neighbor detectors are strongly correlated. Also, the Pearson Correlation Coefficient (PCC) between each Monday's speed data is computed in Table 1. Here, the PCC for vector *x* and *y* is defined as

(1) $$\begin{array}{c} \rho_{x,y}=\frac{\mathrm{cov}\left(x,y\right)}{\sigma_{x}\sigma_{y}}=\frac{E\left(\left(x-E\left(x\right)\right)\left(y-E\left(y\right)\right)\right)}{\sigma_{x}\sigma_{y}}, \end{array}$$

where cov(·) stands for the covariance and *E*(·) stands for the mathematical expectation.

**Figure 3:**
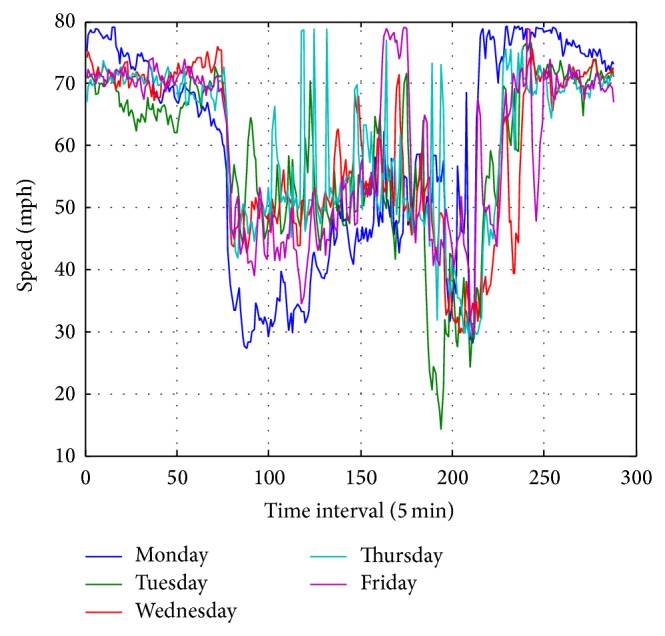
The daily profile of the traffic speed data of five weekdays for VDS 716331.

Besides the strong spatial correlations of traffic speed data, the temporal correlations are also prominent. In order to illustrate the temporal correlations of the traffic speed data, the daily data for five weekdays during a week is plotted in Figure 3. The correlations of the speed data between each weekday are obvious. However, the fluctuations of each data profile are notable; this is the inherent property of traffic speed data especially when the traffic condition is in congestion. Also, the PCCs of the data sets are shown in Table 2.

**Figure 4:**
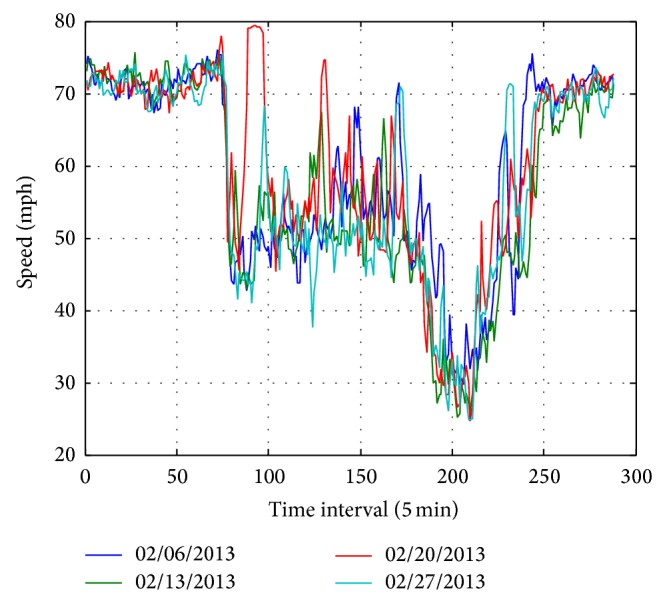
The daily profile of the traffic speed data on Wednesday in February 2013.

It is worth mentioning that the correlations along week-to-week mode should be more prominent. For verifying the idea, the speed data on Wednesday for a month (February 2013) are plotted in Figure 4, and the PCCs are shown in Table 3. The correlations are stronger than those between weekdays.

Simultaneously, the correlations of traffic speed data are of multiple patterns. The interval-to-interval correlations are usually ignored because it may be less apparent than day-to-day correlations. After making statistics and analysis, the PCCs of interval-to-interval pattern are averaged as about 70%. The temporal correlations are not so stronger than traffic volume data. Thus, more accurate methods are needed to develop missing traffic speed data.

According to the above analysis, it is sufficient to say that traffic speed data on a freeway corridor exhibit a strong correlation in multimode. In day mode as well as week mode, space as well as interval mode, PCCs are about 0.7. It should be noted that PCC value can be underestimated and/or misleading if outliers that are ubiquitous in traffic speed data are present [24]. Some robust statistical methods, such as the methods proposed by Verma et al. [25, 26], have been proposed in recent years. These methods would provide more powerful tools for traffic speed data analysis. This will be considered in our future work to help us to understand the intrinsic features underlying the traffic speed data.

## 4. HaLRTC for Traffic Speed Completion

Based on the above correlations analysis of traffic speed data, the tensor model is firstly constructed along different modes. The correlations of traffic speed data are critical for recovering the missing traffic speed data. Traditional methods mostly exploit part of correlations, such as historical or temporal neighboring correlations. The classic methods usually utilize the temporal correlations of traffic speed data from day to day. Such as the single detector data, multiple correlations contain the relations of traffic speed data from day to day, hour to hour, and so forth. In addition, the spatial correlations exist in multiple detectors speed data.

Conventional methods usually use day to day matrix pattern to model the traffic speed data. Although each mode of traffic speed data has a very high similarity, these methods do not utilize the multimode correlations, which are “Day × Hour,” “Week × Hour,” and “Link × Hour,” simultaneously and thus may result in poor recovery performance.

To make full use of the multimode correlations and spatial-temporal information, traffic speed data need to be constructed into multiway data model. Fortunately, tensor pattern based traffic speed data can be well used to model the multiway traffic speed data. This helps keep the original structure and employ enough spatial-temporal information. For example, the speed data set which is used for correlations analysis in Section 3 can be constructed as a 13 × 28 × 24 × 12 tensor model according the PCCs computed along each mode. Here, the speed tensor of size 13 × 28 × 24 × 12, which stands for 13 detectors, 28 days, 24 hours in a day, and 12 sampling intervals in a hour (i.e., sampling interval is 5 min). For imputing the missing speed data, the built tensor model can keep up the integrity of speed data structure and exploit multimode correlations simultaneously. Meanwhile, an efficient algorithm is equally important.

Considering the higher fluctuation characteristics of traffic speed data, a high accuracy low rank tensor completion (HaLRTC) algorithm [17] is used for imputing missing traffic speed data in this paper. In the following, a single detector speed profile is created as a three-order tensor *𝒳* ∈ *ℛ* ^*l*×*m*×*n*^ for expressing simply; also it is the same for multiple detectors speed data. The speed tensor *𝒳* ∈ *ℛ* ^*l*×*m*×*n*^ contains average speed values for *l* days, *m* hours, and *n* intervals. However, not all values in *𝒳* are known. Let *Ω* be the set of values for which speed data is available. Just as the correlations analysis in Section 3, strong temporal and spatial correlations are exhibited. Hence, the speed data can be represented as a low-dimensional structure. Thus, the problem of imputing missing traffic speed data can be solved by the optimization problem for low rank tensor completion:

(2) min⁡X:⁡ rank(X)  s.t.: XΩ=TΩ,

where *𝒳*, *𝒯*, are *n*-mode tensors with identical size in each mode. However, the rank of tensor is not unique and nonconvex [27]. One common approach is to use the trace norm ‖·‖_∗_ which is the tightest convex envelop for the rank of tensors to approximate the rank of tensors. Using the definition of trace norm of tensor in [17], the optimization problem can be converted into

(3) min⁡X:⁡ ∑i=1nαiX(i)∗  s.t.: XΩ=TΩ,

where *α*_*i*_'s are constants satisfying *α*_*i*_ ≥ 0 and ∑_*i*=1_^*n*^ *α*_*i*_ = 1. To solve the optimization problem, additional tensors *ℳ*_1_, …, *ℳ*_*n*_ are introduced and derive the following equivalent formulation:

(4) min⁡X,M1,…,Mn:⁡ ∑i=1nαiMi(i)∗   s.t.: X=Mi for  i=1,…,n     XΩ=TΩ.

In this paper, a high accuracy algorithm called ADMM [28] is introduced to tackle the large scale problem. The augmented Lagrangian function of (4) is as follows:

(5) Lρ(X,M1,…,Mn,Y1,…,Yn) =∑i=1nαiMii∗+X−Mi,Yi   +ρ2Mi−XF2.

According to the framework of ADMM and the algorithm description in [17], the HaLRTC algorithm is summarized in Algorithm 1.

## 5. Experiments

The performances of HaLRTC algorithm are evaluated on the real world traffic speed data. The experimental settings are listed in Section 5.1 and evaluation indices are shown in Section 5.2. In Section 5.3, temporal correlations are exploited for imputing missing speed data for a single detector. Additionally, Section 5.4 tests the algorithms for data sets of multiple detectors.

### 5.1. Experimental Settings

HaLRTC is compared with two classical imputation methods: (1) Mean-Historical imputation [29] and (2) BPCA-based imputation method [10].

For the historical imputation method, we calculate the mean value of all the available data points belonging to the same detector at the same time interval in the last few days and use the mean as the imputed value; see [29] for more details. For BPCA, we set the maximum number of iteration steps is 200 and the threshold of the approximate complexity is set to 10^−4^ which is the same as [10]. HaLRTC method is an iterative algorithm; the maximum iterative numbers are set as 500. The value of *α*_*i*_ is set to 1/*n* (*n* is the mode number), and the parameter *ρ* is set as *e* ^−3^.

### 5.2. Evaluation Indices

To evaluate the performances of the proposed method HaLRTC, the following two indices were used in this paper.

*(1) Mean Absolute Percentage Error (MAPE)*. The index gives the evaluation of the average estimation error in terms of percentage:

(6) $$\begin{array}{c} \text{MAPE}=\frac{1}{M}\overset{M}{\underset{m=1}{\sum }}\left|\frac{t_{r}^{\left(m\right)}-t_{e}^{\left(m\right)}}{t_{r}^{\left(m\right)}}\right|\times 100. \end{array}$$

*(2) Root Mean Square Error (RMSE)*. This index gives the evaluation of the variance in the estimation errors:

(7) $$\begin{array}{c} \text{RMSE}=\sqrt{\frac{1}{M}\overset{M}{\underset{m=1}{\sum }}{\left(t_{r}^{\left(m\right)}-t_{e}^{\left(m\right)}\right)}^{2}}, \end{array}$$

where *t*_*r*_^(*m*)^ and *t*_*e*_^(*m*)^ are the *m*th elements which stand for the known real value and estimated value, respectively. *M* denotes the number of estimated traffic volumes.

### 5.3. Results for Missing Speed Data of Single Detector

To illustrate the performances of the proposed method, a complete traffic speed data set is used as ground truth for the test. We choose the data of a fixed detector VDS 716331 in District 7, Los Angeles County (see Figure 1) which are downloaded from (PeMS: http://pems.dot.ca.gov/). The traffic speed data are recorded every 5 minutes. Therefore, a daily traffic speed series for a loop detector contains 288 records, and the whole period of the data is chosen for three weeks, that is, from February 4 to February 24, 2013.

Based on the correlations analysis of traffic speed data in Section 3, the correlations are stronger for weekdays without regard to the weekends. And it will be helpful for imputing the missing traffic speed data. Therefore, the weekdays' data for three weeks (i.e., 15 days) are chosen for evaluating the HaLRTC algorithm.

Based on multiple correlations of the traffic speed data, the data set is modeled as a tensor of size 24 × 12 × 15 which stands for 24 hours in a day, 12 sample intervals (i.e., recorded by 5 minutes) per hour, and 15 days. For the methods Mean and BPCA, the speed data is arranged as a matrix of size 288 × 15. The ratios of missing data are set from 5% to 80% and the missing data are produced randomly. All the results are average by 10 instances.

**Figure 5:**
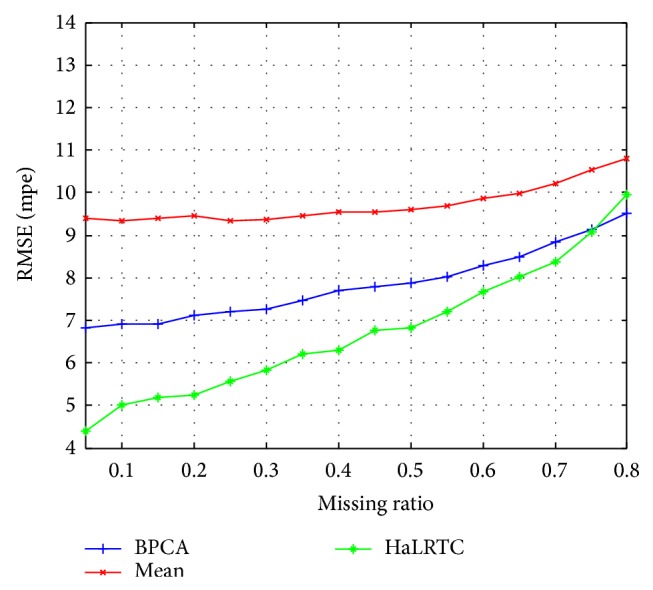
RMSE for weekdays' data of single detector.

**Figure 6:**
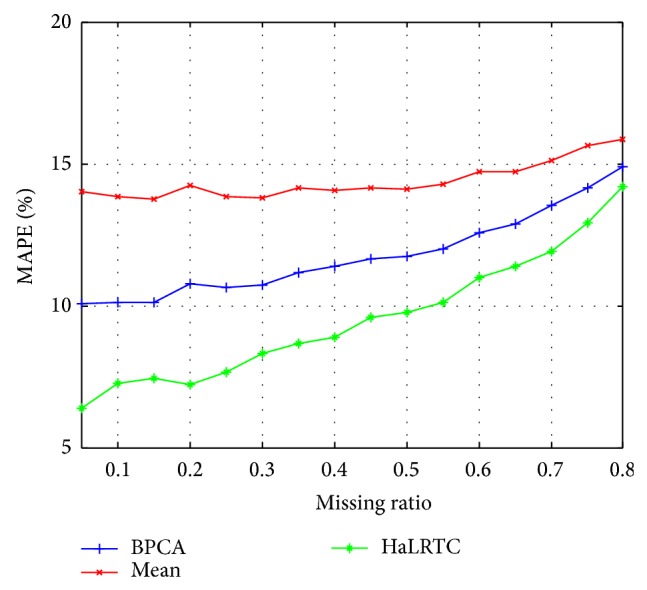
MAPE for weekdays' data of single detector.

The RMSE curves and MAPE curves of those methods with randomly missing weekdays' traffic speed data for a single loop detector are shown in Figures 5 and 6. Obviously, the RMSE and MAPE of HaLRTC approach are smaller than other approaches. It is worth noting that the RMSE of HaLRTC will be bigger than RMSE of BPCA when the missing ratio is 80%. That situation can be considered as a diploma when the HaLRTC will reach the limit for keeping the high accurate.

For exploiting stronger correlations of traffic speed data, Wednesday's data of a specific week are chosen, and 10 weeks data make up a data set with 10 days. Identically, the data set is modeled as a tensor of size 24 × 12 × 10 which stands for 24 hours in a day, 12 sample intervals (i.e., recorded by 5 minutes) per hour, and 10 days. And a 288 × 10 matrix model is arranged for Mean and BPCA.

**Figure 7:**
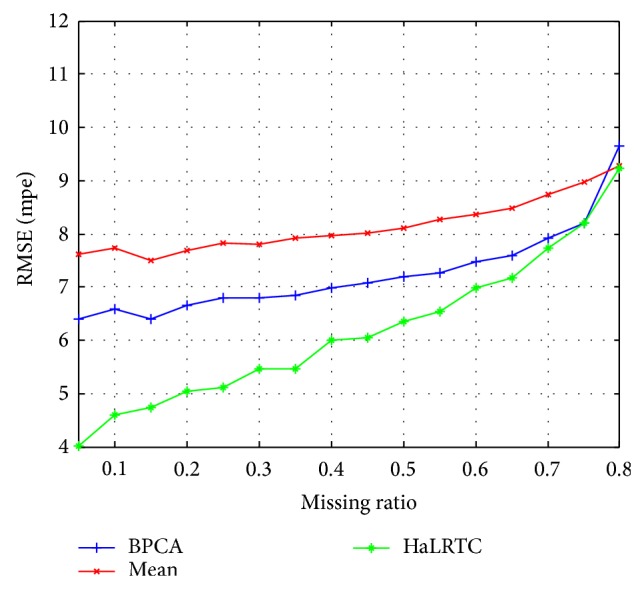
RMSE for data in Wednesday of single detector.

**Figure 8:**
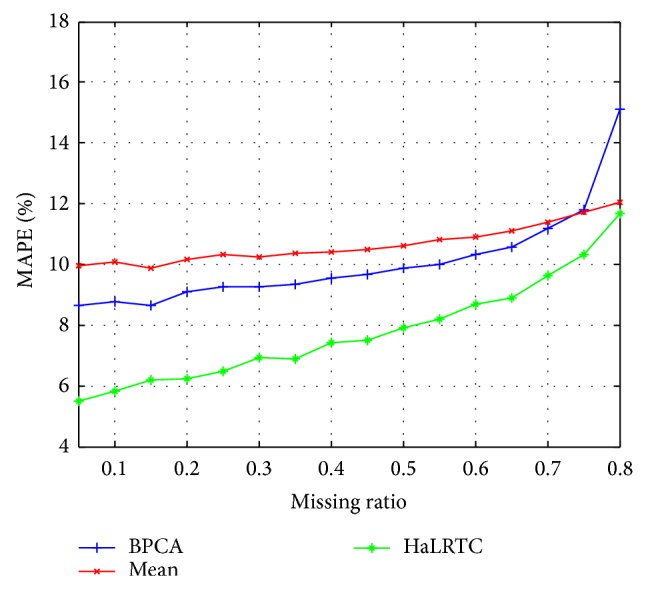
MAPE for data in Wednesday of single detector.

Figures 7 and 8 show that HaLRTC algorithm is with higher accuracy for imputing missing traffic speed data than Mean and BPCA. Comparing Figures 5 and 7 corresponding with comparison of Figures 6 and 8, the performance for imputing missing traffic speed data will better when exploiting more correlations of data sets.

### 5.4. Results for Missing Speed Data of Multiple Detectors

To illustrate the benefit of HaLRTC algorithm in reconstructing the missing traffic speed data, that is, multiple correlations of traffic speed data are more beneficial than the partial correlations used in traditional methods. A new traffic speed data set by considering spatial correlations is used to evaluate HaLRTC algorithm.

We choose the data of 13 detectors in District 7, Los Angeles County (see Figure 1) which are downloaded from (PeMS: http://pems.dot.ca.gov/). For each detector, the traffic speed data for a specific day are chosen and derive the data set. For exploiting the spatial and temporal correlations of traffic speed data simultaneously, the data set is modeled as a tensor of size 24 × 12 × 13, which stands for 24 hours in a day, 12 sampling intervals in an hour, and 13 detectors. Meanwhile, the data set is arranged as a matrix 288 × 13 for Mean and BPCA.

**Figure 9:**
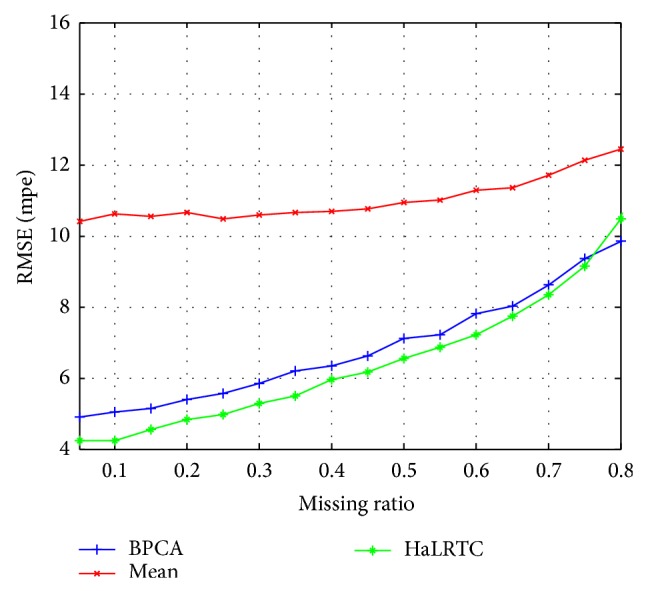
RMSE for data of multiple detectors.

**Figure 10:**
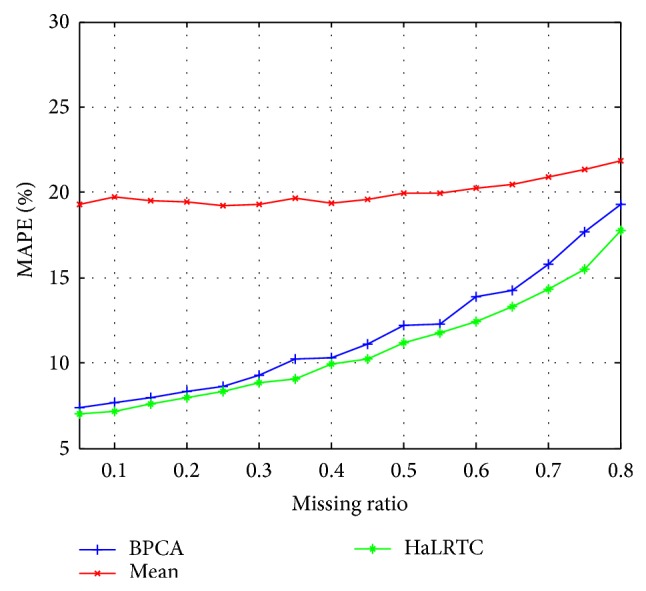
MAPE for data of multiple detectors.

Figures 9 and 10 show that our propose HaLRTC outperforms Mean and BPCA for traffic speed data of multiple detectors.

## 6. Conclusion

In this paper, a multiway tensor model is proposed to represent the traffic speed data considering the multiple correlations, and a high accuracy low rank tensor completion (HaLRTC) algorithm is employed to estimate the missing traffic speed data due to its severely fluctuation. Experiments on benchmark show that the proposed method performs better than other classical state-of-the-art methods. For future work, it is interesting to extend the proposed method to large and dynamic road network.

## Figures and Tables

**Algorithm 1:**
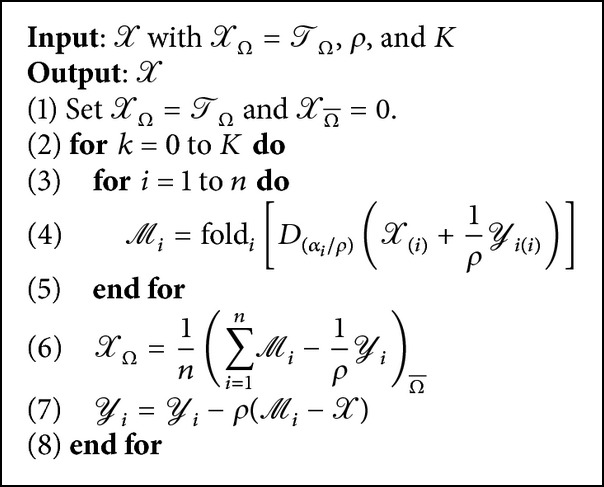
HaLRTC: high accuracy low rank tensor completion.

**Table 1:** Pearson Correlation Coefficient between five weekdays for VDS 716331.

PCC	Link1	Link2	Link3	Link4	Link5	Link6	Link7
Link1	1.0000	0.9617	0.9454	0.8255	0.8698	0.7567	0.7187
Link2	0.9617	1.0000	0.9660	0.8735	0.8584	0.8012	0.7564
Link3	0.9454	0.9660	1.0000	0.9233	0.9247	0.8521	0.7972
Link4	0.8255	0.8735	0.9233	1.0000	0.8507	0.9526	0.8982
Link5	0.8698	0.8584	0.9247	0.8507	1.0000	0.8325	0.7898
Link6	0.7567	0.8012	0.8521	0.9526	0.8325	1.0000	0.9694
Link7	0.7187	0.7564	0.7972	0.8982	0.7898	0.9694	1.0000

**Table 2:** Pearson Correlation Coefficient between five weekdays for VDS 716331.

PCC	Monday	Tuesday	Wednesday	Thursday	Friday
Monday	1.0000	0.6082	0.6184	0.6286	0.7261
Tuesday	0.6082	1.0000	0.7757	0.7124	0.6882
Wednesday	0.6184	0.7757	1.0000	0.8264	0.7382
Thursday	0.6286	0.7124	0.8264	1.0000	0.6609
Friday	0.7261	0.6882	0.7382	0.6609	1.0000

**Table 3:** Pearson Correlation Coefficient on Wednesday in February 2013.

PCC	Monday	Tuesday	Wednesday	Thursday
Monday	1.0000	0.8733	0.7656	0.8738
Tuesday	0.8733	1.0000	0.8422	0.8890
Wednesday	0.7656	0.8422	1.0000	0.8139
Thursday	0.8738	0.8890	0.8139	1.0000
